# Dengue Surveillance among French Military in Africa

**DOI:** 10.3201/eid1802.111333

**Published:** 2012-02

**Authors:** Franck de Laval, Sébastien Plumet, Fabrice Simon, Xavier Deparis, Isabelle Leparc-Goffart

**Affiliations:** Epidemiologic and Public Health Military Center, Marseille, France (F. de Laval, X. Deparis);; French Army Forces Biomedical Institute, Marseille (S. Plumet, I. Leparc-Goffart);; Laveran Military Hospital, Marseille (F. Simon)

**Keywords:** dengue fever, dengue virus, virus, serotype, Africa, epidemiologic surveillance, French Armed Forces, military

**To the Editor:** In their recent article, Amarasinghe et al. (*1*) describe dengue virus distribution in Africa. Their data were based on published reports of dengue cases among local populations and travelers returning from Africa. To complement the description by Amarasinghe and colleagues of dengue serotypes found in Africa, we report results from dengue virologic testing during 1998–2010. The tests were performed at the Arbovirus National Reference Center (Tropical Medicine Institute of the Military Health Service, Marseille, France).

Each year, ≈14,000 French soldiers are stationed in dengue-endemic areas of Africa (mainly Cameroon, Central African Republic, Chad, Djibouti, Gabon, Côte d’Ivoire, Senegal, and Mayotte and Reunion islands), from which they travel throughout Africa. The population of soldiers is under constant epidemiologic surveillance. If symptoms of dengue fever develop in a soldier, a blood sample and a dengue-specific questionnaire from the patient are sent to the Tropical Medicine Institute of the Military Health Service. Virus culture and reverse transcription PCR, or both, were performed on early samples; otherwise, serologic testing was performed by using in-house assays (IgM antibody capture ELISA and direct IgG ELISA).

During the 12 years of surveillance, the laboratory received 2,423 samples from patients with suspected dengue within the French Armed Forces in Africa. Of these, 224 were probable acute dengue infections: 202 had positive IgM serologic results for dengue, and 22 were confirmed as dengue cases by RT-PCR or culture (Table). Serologic data may be confusing because of potential cross-reactions with other flavivirus antibodies (in particular in Chad with West Nile virus).

**Table:** Countries in Africa with evidence of dengue virus transmission among French Armed Forces, 1998–2010

Country and year	No. cases	Testing method	Infection status	Dengue virus serotype
Cameroon, 2010	1	PCR	Confirmed	1
Cape Verde, 2010	5	Culture	Confirmed	3
Central African Republic, 1995	1	Serology	Probable	Unknown
Chad, 1998–2001, 2003, 2006, 2009–2010	28	Serology	Probable	Unknown
Comoros				
2010	1	PCR, culture	Confirmed	1
2010	2	PCR	Confirmed	3
Côte d’Ivoire				
1999	1	Culture	Confirmed	1
2000, 2004–2007	11	Serology	Probable	Unknown
2010	1	PCR	Confirmed	3
Djibouti				
1998	4	Culture	Confirmed	1
1998	24	Serology	Probable	Unknown
2000	2	Culture	Confirmed	1
2000	4	Serology	Probable	Unknown
2001–2005	123	Serology	Probable	Unknown
2005	1	PCR	Confirmed	ND
2006	4	Serology	Probable	Unknown
2008	2	Serology	Probable	Unknown
Gabon				
1998, 2006–2008	22	Serology	Probable	Unknown
2010	1	PCR	Confirmed	1
Mayotte, 2009	1	Culture	Confirmed	1
Senegal, 2009	1	PCR	confirmed	3
Somalia, 1999	1	culture	confirmed	2

Because of probable underreporting from the field, our reported number of confirmed dengue cases likely underestimates the actual number of cases among French troops stationed in Africa. Nonetheless, our data complement those reported by Amarasinghe et al. by demonstrating additional locations for circulation of serotype 1 (Cameroon, Djibouti, Gabon, Mayotte) and serotype 3 (Comoros). Military epidemiologic surveillance systems can detect dengue circulation where soldiers stay. Thus, these systems could serve to evaluate the risk for dengue infection in countries without local epidemiologic surveillance systems, thereby improving knowledge about dengue circulation in African countries.
